# The Role of Innate Immunity Receptors in the Pathogenesis of Inflammatory Bowel Disease

**DOI:** 10.1155/2015/936193

**Published:** 2015-03-04

**Authors:** Paula Peruzzi Elia, Yolanda Faia M. Tolentino, Claudio Bernardazzi, Heitor Siffert Pereira de Souza

**Affiliations:** ^1^Serviço de Gastroenterologia and Laboratório Multidisciplinar de Pesquisa, Hospital Universitario, Universidade Federal do Rio de Janeiro, 21941-913 Rio de Janeiro, RJ, Brazil; ^2^D'Or Institute for Research and Education (IDOR), Rua Diniz Cordeiro 30, Botafogo, 22281-100 Rio de Janeiro, RJ, Brazil

## Abstract

Innate immunity constitutes the first line of defense, fundamental for the recognition and the initiation of an inflammatory response against microorganisms. The innate immune response relies on the sensing of microbial-associated molecular patterns through specialized structures such as toll-like receptors (TLRs) and the nucleotide oligomerization domain- (NOD-) like receptors (NLRs). In the gut, these tasks are performed by the epithelial barrier and the presence of adaptive and innate immune mechanisms. TLRs and NLRs are distributed throughout the gastrointestinal mucosa, being more expressed in the epithelium, and in lamina propria immune and nonimmune cells. These innate immunity receptors exhibit complementary biological functions, with evidence for pathways overlapping. However, as tolerance is the predominant physiological response in the gastrointestinal mucosa, it appears that the TLRs are relatively downregulated, while NLRs play a critical role in mucosal defense in the gut. Over the past two decades, genetic polymorphisms have been associated with several diseases including inflammatory bowel disease. Special emphasis has been given to the susceptibility to Crohn's disease, in association with abnormalities in the NOD2 and in the NLRP3/inflammasome. Nevertheless, the mechanisms underlying innate immune receptors dysfunction that result in the persistent inflammation in inflammatory bowel disease remain to be clarified.

## 1. Introduction

In the gastrointestinal system, homeostasis represents a rather complex and dynamic process, with a critical role for mucosal immunity. In physiological conditions, it is expected that the host identifies and responds appropriately to the luminal contents of the gastrointestinal tract. In this regard, the epithelium, constituted by a single cell lining, plays an important role separating an essentially sterile internal milieu from a formidable burden of microbes that populate the gastrointestinal tract [1]. In conjunction with the epithelium, the intestinal immune system also has a critical challenge of distinguishing commensal from pathogenic microorganisms, in a complex and yet incompletely understood mechanism [2].

The interaction between the gut and the microorganisms that constitute the resident microbiota is tightly regulated and has evolved in the course of several million years [3]. In fact, this mutualistic relationship between host and microbiota is thought to be essential for the immune homeostasis and is well balanced in normal conditions [4, 5]. However, its disequilibrium has been implicated in the development of various diseases, including inflammatory bowel disease (IBD) and its two major forms, Crohn's disease (CD) and ulcerative colitis (UC) [6–8]. In this paper, we are going to present an overview of the basic mechanisms of the innate immunity and the defects associated with the development of IBD.

## 2. Innate Immunity in the Intestine

The innate immune system represents the first line of defense against invading microorganisms and is critically important in the early recognition and subsequent initiation of an inflammatory response [9]. In contrast to the adaptive immunity, the response mounted by the innate immune system has been regarded as relatively nonspecific, being mediated primarily by macrophages, dendritic cells, and granulocytes, basically functioning as phagocytes and antigen presenting cells [10]. The innate immune response depends on the recognition of evolutionarily conserved structures expressed on microbes, the microbial-associated molecular patterns (MAMPs), through special cell receptors.

**Figure 1:**
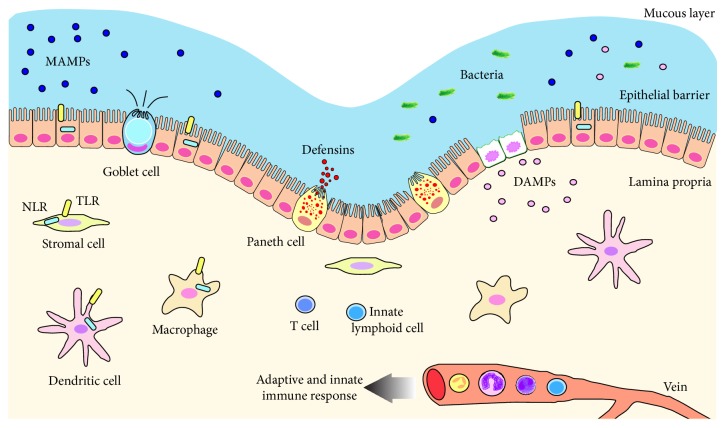
The mechanism of intestinal response against MAMPs and DAMPs in normal conditions. The epithelial barrier recognizes microbial-associated molecular patterns (MAMPs) by the presence of transmembrane TLRs and intracellular microbes and damage-associated molecular patterns (DAMPs), by the cytosolic NLRs. When invading the lamina propria, microorganisms can be recognized through the same mechanisms, by other cells such as dendritic cells, macrophages, lymphocytes, innate lymphoid cells, and stromal cells. The result of the activation of immune cells in the lamina propria and the degree of cell damage, caused by chemokines and cytokines, determine the feedback of the system. TLRs and NLRs drive the immune response and contribute to the maintenance of homeostasis.

In order to control the number and composition of microbial populations and also to identify potential pathogens, the host needs to maintain surveillance over the microbiota. In the gastrointestinal tract, these tasks are performed by the epithelial barrier and the presence of adaptive and innate immune mechanisms [11, 12] (Figure 1).

**Figure 2:**
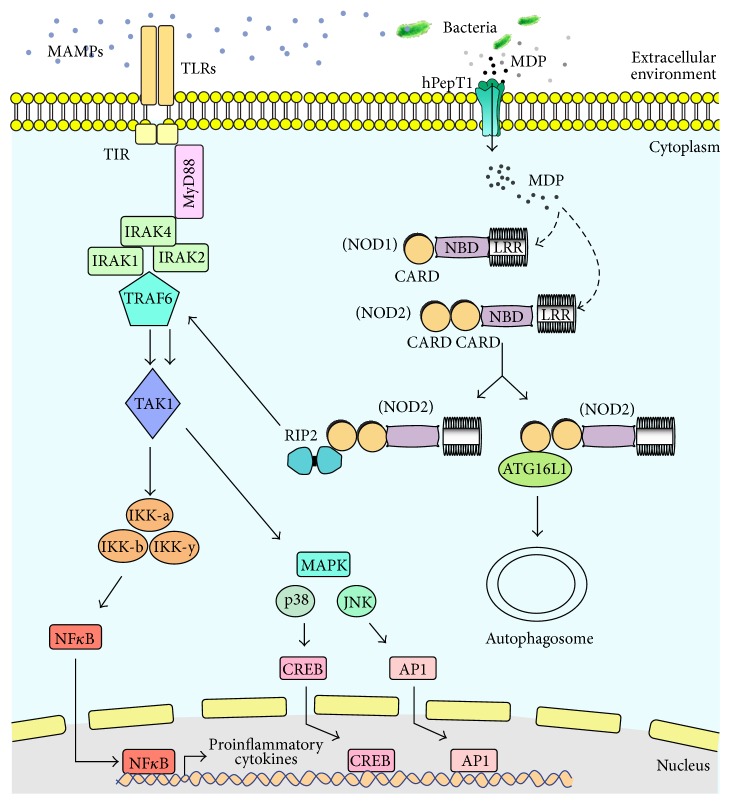
TLR and NLR pathways. The TLR pathway is composed of the conserved domain toll-IL-1-resistence (TIR), which senses microbial-associated molecular pattern (MAMPs) and interacts with the myeloid differentiation primary-response protein 88 (MyD88). MyD88 drives signaling through NF-kappa B, by interacting with the IL-1R-associated kinases 1, 2, and 4 (IRAK1, 2, and 4), TNF receptor-associated factor 6 (TRAF6), TGF-*β* activated kinase 1 (TAK1), and the inhibitor of kappa B (IKKa, b, and y), promoting the activation of proinflammatory cytokines (left). The NLR pathway can be activated by bacterial muramyl dipeptide (MDP), interacting with the leucine-rich repeat-containing protein (LRR) present in NOD1 and NOD2 structures. Both NOD1 and NOD2 can interact with the adaptor molecule RICK (RIP2) via caspase recruitment domains (CARD-CARD) and stimulate TRAF6, which drives the activation of other elements of NF-kappa B and MAPK pathways, with the consequent production of proinflammatory cytokines (right). Additionally, NOD2 can interact with ATG16L1 and stimulate the formation of the autophagosome.

Interactions of the epithelium and other innate immunity cells with microbes are mediated by the presence of transmembrane or cytosolic receptors, called pattern recognition receptors (PRRs), capable of sensing and recognizing specific microbial compounds known as MAMPs [13]. In fact, not only whole microbes, but also diffusible components can interact with the PRRs. These signaling receptors comprise at least three distinct families: toll-like receptors (TLRs), the nucleotide oligomerization domain (NOD-) like receptors (NLRs), and retinoic acid inducible gene I- (RIG-I-) like receptors (RLRs) [14]. Among these receptors, the NOD-like receptors (NLRs) protect the intracellular cytosolic compartment, while the transmembrane toll-like receptors (TLRs) survey the extracellular space [14]. Upon MAMPs recognition, these innate receptors recruit adaptor proteins and cellular kinases, which in turn trigger distinct intracellular signaling cascades, culminating in the activation of the MAPK and NF-kappa B pathways [14, 15] (Figure 2).

## 3. Toll-Like Receptors in the Intestine

Currently, the TLR family is the best characterized in mammals and is composed of 13 receptors [11]. MAMPs sensing and specificity associated with TLRs are achieved through the arrangement and sequence variation in the conserved leucine-rich repeat (LRR) domains. TLRs are localized in the cell membrane and/or endosomal membrane components and are able to recognize extracellular and endocytosed ligands. For example, lipopolysaccharide internalization was shown to be required for chemokine induction, supporting the idea that NF-kappa B activation might depend upon intracellular TLR4 signaling within the epithelium [16].

In the human gastrointestinal tract, most TLRs have been shown to be present, with some particularities in terms of distribution and function [17]. TLR5 is basically expressed in the colonic epithelium and recognizes invasive flagellated bacteria, while TLR2 and TLR4 are present in low levels in the intestinal epithelium, more abundantly in the colonic crypts [18]. On the other hand, TLR3 appears to be predominantly expressed in mature enterocytes in both the small bowel and the colon [14, 19]. Interestingly, in regard to TLR9 in the intestinal epithelium, it has been demonstrated that activation through the apical membrane determines tolerance, while through the basal membrane it induces activation of the canonical NF-kappa B pathway [20]. The differential spatial distribution of TLR in the epithelial cells reinforces the role of PRR signaling in innate immunity and may constitute a critical regulatory mechanism to distinguish commensal microbiota from pathogens.

## 4. NOD-Like Receptors and Inflammasome in the Intestine

The NLRs have been shown to play a key role in the defense against intracellular microbes, being capable of recognizing a broad range of exogenous bacterial components and toxins, as well as certain endogenous damage-associated molecular patterns (DAMPs) [21]. The NLR family comprises more than twenty cytosolic receptors in mammals, divided in different groups based on the N-terminal activation domains involved in signal transduction [14]. All these domains have been implicated in the triggering of alternative signaling pathways, including caspase and NF-kappa B activation, leading to the expression of inflammatory mediators and defensins, and the regulation of apoptotic signals [22].

Among the NLRs that recognize microbial molecules derived from peptidoglycan metabolism, only the NOD1 and NOD2 functions have been well characterized in the gastrointestinal tract. While NOD1 senses the dipeptide g-D-glutamyl-meso-diaminopimelic acid (iE-DAP) [23, 24] originated from most Gram-negative and also specific Gram-positive bacteria [25], NOD2 recognizes muramyl dipeptide (MDP), a ubiquitous component of all types of peptidoglycans [24].

In regard to tissue distribution, NOD1 receptors are constitutively expressed in a wide range of cells of both the hematopoietic and nonhematopoietic lineage, including intestinal epithelial cells [26–28]. On the other hand, NOD2 expression has been reported primarily in hematopoietic cells, particularly in APC. Notably, in the epithelial compartment, NOD2 appears to be restricted to Paneth cells in the small bowel [29]. Nevertheless, upon exposure to inflammatory stimuli, such as TNF-alpha and IFN-gamma, NOD2 expression has been shown to become upregulated [30].

Although the exact biological role of NOD1 and NOD2 in the intestinal innate immunity is yet to be determined, it has been suggested that TLR and NOD1 or NOD2 may act in a complimentary fashion in regard to specific microbes. Because TLR signaling is downregulated within the intestine, in order to avoid continuous inflammation induced by the commensal microbiota, it is reasonable to suppose that NOD1 and NOD2 would then play a critical role in the host defense. Of note, both NOD1 and NOD2 may perform highly specialized and essential antimicrobial functions, such as regulation of antimicrobial peptides, therefore being critically important at mucosal surfaces [31, 32].

In contrast to NOD1 and NOD2 stimulation, which are involved primarily in activation of inflammatory pathways, signaling through other NLR proteins results in activation of caspases. As a consequence of this NLR signaling, pro-caspase-1 is recruited to a multiprotein complex known as inflammasome [33], composed of an NLR family member, such as NLRC4 (previously known as Ipaf, Ice protease-activating factor), NLRP (NAcht LRR protein) 1, or NLRP3/Cryopyrin, and the adaptor ASC (apoptosis-associated speck-like protein containing a CARD) [33, 34]. Oligomerization of these subunits through multiple complex molecular interactions results in the activation of caspase-1, which in turn catalyzes the cleavage of inactive IL-1 beta precursor, accumulated in the cytosol, determining the maturation of the inflammatory cytokines IL-1 beta and IL-18 [33, 35, 36].

**Figure 3:**
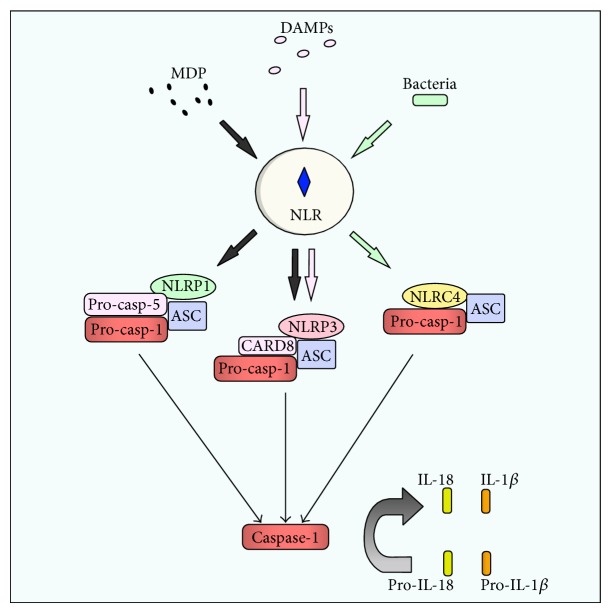
The inflammasome pathway. Depending on the type of stimulus and the type of cell or tissue, signaling through NLR proteins can activate different inflammasomes. Inflammasome is a multiprotein complex composed of NACHT LRR protein (NLRP) and apoptosis-associated speck-like protein containing CARD (ASC), which cleaves procaspase-1 (pro-casp-1) in caspase-1. Once activated, caspase-1 catalyzes the cleavage of pro-IL-18 and pro-IL-1*β* into IL-18 and IL-1*β*, respectively, promoting the inflammatory response.

Specific inflammasome subtypes have been described, according to their respective NLR, each recognizing distinct MAMPs or other danger signals [37]. For instance, NLRP1 and NLRP3 have been shown to trigger caspase-1 activation in response to bacterial MDP [38, 39]. On the other hand, additional roles have been described for NLRP3, which also recognizes viral RNA [40] and bacterial DNA [41], and also potential DAMPs, such as ATP [34] and uric acid crystals [42] (Figure 3).

## 5. Defective Innate Immunity in IBD

The pathogenesis of IBD has been regarded as multifactorial in origin, encompassing genetic susceptibility, epithelial barrier dysfunction, and an abnormal immune response to luminal contents, however, with an increasingly recognized role for innate immunity defects in the last ten years [7, 43].

### 5.1. TLR Abnormalities in IBD Epithelium

Most data on the role of TLR in the intestinal epithelium have derived from studies with experimental models and cell lines. In respect of the role of TLR in the human intestinal epithelium, investigations have yielded considerable heterogeneous results. In primary human epithelial cells, obtained from intestinal samples, the expression of TLR-2 and TLR-4 has been quite variable, being described at the crypts [44, 45], in low levels [46] or even completely absent [47]. However, in mucosal samples from patients with IBD, epithelial TLRs were reported to be absent [47] or overexpressed for TLR-4 [46]. More recently, enhancement of both TLR2 and TLR4 in colonic crypt epithelial cells isolated from mucosal tissue has been demonstrated in patients with IBD [48]. In regard to TLR5, it is noticeable that it is not widely expressed outside the gastrointestinal tract [49]. However, interestingly, its ligand flagellin is reported as a dominant epitope in sera from IBD patients [50, 51], whereas it appears to trigger a cytoprotective effect in the gastrointestinal tract [52, 53]. In experimental animals, TLR5 deficient mice have been demonstrated to develop spontaneous colitis [54], supporting the suggested protective role of TLR5 in humans.

Taken together, the findings regarding TLR expression and function in IBD, so far, suggest that colonic crypt epithelial cells may have a greater capacity to respond to stimuli derived from the intestinal microbiota.

### 5.2. NLR Abnormalities in IBD

After the discovery of the association of NOD2 polymorphisms with CD, in the last decade IBD has progressively been positioned right at the forefront of the new genome-wide association studies (GWAS) era. A number of GWAS have attempted to find inherited elements of IBD, with a successful identification of more than 160 loci [55, 56]. However, these studies fail to explain most of IBD associated heritability and have been directed to limited populations [57], while IBD is spreading all over the world [58].

In regard to NLRs, they are known to display a broad expression throughout the body, and the altered expression of these molecules in the intestinal tissues has been shown to be associated with the pathogenesis of intestinal inflammation, in both humans and experimental models [59].

#### 5.2.1. NOD2 and IBD

For more than a decade, a defective* NOD2* gene (also termed caspase recruitment domain family, member 15,* CARD15*) has been known to constitute the most common genetic defect associated with CD [60, 61]. Of note, the CD-associated* NOD2* gene polymorphisms determine a loss-of-function in the NOD2 pathway [62]. Although it is well established that NOD2 activation elicits acute signaling effects, other diverse cellular modifications also appear to be relevant to the immune response and the intestinal homeostasis [63]. Typically, stimulation with MDP induces NOD2 oligomerization through the central NACHT domain and binding of the RIP2 kinase through CARD-CARD interactions [64]. The NOD2-RIP2 complex then initiates a signaling cascade with potentially multiple outcomes, such as the activation of the IkB kinase (IKK) complex and MAPKs activation, with the consequent expression of cytokines, chemokines, and antimicrobial peptides [65], autophagy and resistance to intracellular microorganisms [66], and the modulation of antigen expression through the major histocompatibility complex [67] (Figure 2).

Currently, it remains to be elucidated how the loss-of-function polymorphisms on NOD2 signaling determines the risk for CD development. Nevertheless, it has been proposed that decreased NOD2 function results in a defective interaction between the mucosal immune system and the intestinal microbiota, with an abnormal response to pathogens, potential bacterial invasion, and persistent intestinal inflammation [63]. It is intriguing to notice, however, that downstream NOD2 signaling dysfunction can be detected, even in the majority of CD patients who do not display NOD2 polymorphisms. This evidence suggests a relatively limited participation of NOD2 in CD, but it also indicates an ambiguous role of the receptor in the pathogenesis of chronic intestinal inflammation.

As NOD2 signaling emerges as a key regulator of NF-kappa B activation and the consequent induction of proinflammatory cytokines, it also has a critical role in mucosal protection. On the other hand, the expressions of NOD2 per se, together with the proinflammatory cytokines, increase substantially also as a result of inflammatory stimuli [68]. In fact, this complex and bidirectional function of NOD2 appears to be dependent on the stage of the inflammatory disease [69]. For example, it has been shown that children with CD display an overexpression and hyperactivity of NOD2 and RIP2, its obligate caspase-recruitment domain-containing kinase, in biopsy samples from the intestinal inflamed mucosa [70]. However, while NOD2-deficient mice do not develop intestinal inflammation spontaneously, they were shown to be more susceptible to microbial infection, particularly through the oral route [31]. Furthermore, in another IBD experimental model, IL-10-deficient mice did not develop colitis when NOD2 gene deletion was simultaneously introduced into these mice [71].

Other studies addressed additional roles for NOD2, through the analysis of its interaction with TLRs. For example, in an experimental study using NOD2-deficient mice, investigators demonstrated that NOD2 signaling blocks the TLR2-mediated NF-kappa B activation. Hence, this result is consistent with the notion that NOD2 mutations might be implicated in CD pathogenesis, by leading to an excessive Th1-type of immune response [72]. In an attempt to understand NOD2 modulation on responses to PAMPs, peripheral blood monocytes were exposed to bacterial MDP components and then stimulated with MDP and LPS. Pretreatment with MDP led to a selective tolerance in response to subsequent NOD2 + TLR4 stimulation, suggesting that NOD2 and TLR4 signaling pathways probably converge [73].

Currently, it appears that the understanding of NOD2 functions is still incomplete, especially after the identification of susceptibility variants related to autophagy in CD. In fact, NOD2 and autophagy genes share various similar functions. Because autophagy has been implicated in cellular homeostasis and also in the immune response, through the removal of cell debris and bacterial elements [74], it is reasonable to suppose its potential in the pathogenesis of CD. In particular,* ATG16L1* (autophagy-related 16-like 1) gene polymorphisms have been consistently associated with CD in GWAS [75, 76]. Interestingly, interaction between NOD2 and autophagy genes has been demonstrated recently. In human epithelial cells, NOD2 stimulation with MDP was shown to activate autophagy and microbial elimination, in a ATG16L1- and NOD2-dependent manner, but the response was impaired by CD-associated NOD2 variants [77].

#### 5.2.2. NOD1 and IBD

NOD1 (also known as CARD4) exhibits a similar structure compared with NOD2, except for the amino-terminal domain, consisting of a single CARD [24]. Upon exposure to ligands mostly present in Gram-negative bacteria, NOD1 undergoes a conformational modification that initiates a signaling cascade that culminates with the activation of NF-kappa B and MAPK pathways and inflammatory responses [78] (Figure 2). Although NOD1 receptor has been considered as a candidate factor for susceptibility to IBD, data on* NOD1* gene polymorphisms from different studies have provided conflicting results [79, 80].

#### 5.2.3. Inflammasome-Related NLRs and IBD

Among the four types of inflammasomes described so far, the NLRP3 has been the more consistently associated with CD susceptibility [81, 82]. The NLRP3/cryopyrin protein encoded by the* NLRP3* gene is part of the NLRP3-inflammasome, which constitutes a multimeric platform implicated in caspases activation and the consequent cleavage and secretion of IL-1 beta and IL-18 proinflammatory cytokines [33].

Polymorphisms of the* NLRP3* gene have been linked to CD, but the association has been controversial. For example, NLRP3 SNPs have been associated with lower expression of NLRP3 mRNA and low levels of IL-1 beta in peripheral blood cells and monocytes of CD patients [81]. In a different population with different genetic background, susceptibility to CD was also related to a NLRP3 polymorphism. In contrast to the previous study, investigators reported a gain-of-function polymorphism, proposing a different mechanism, which consists of the induction of caspase-1 activity and the resultant overproduction of IL-1 beta [82]. However, the association between NLRP3 gene and susceptibility to IBD has been questioned, after a GWA study analyzing a different population [83].

In consonance with the importance of defects of NLRP3 for the development of intestinal inflammation, studies analyzing its downstream molecules such as IL-18 confirmed the association with the increased susceptibility to CD [84]. In addition, in sites of active intestinal inflammation in CD, IL-18 [85] and IL-1 beta [86] were shown to be overexpressed.

Despite the controversial results regarding the association of NLRP3 with IBD, the complex mechanisms involved in NLRP3-inflammasome began to be clarified in recent years. For example, pannexin-1, a transmembrane hemichannel associated with the purinergic receptor P2X7, has been proposed to function upstream of NLRP3, as it has been shown to mediate the passage of microbial molecules into the cytosol, triggering NLRP3-inflammasome activation [87]. Moreover, as demonstrated by our group, the site-specific expression and modulation within the gut and gut-associated lymphoid tissues [88] and the upregulation of the P2X7 receptor in an inflammatory microenvironment [89], together with the induction of epithelial cell apoptosis and autophagy by its ligand ATP [90], point to purinergic signaling as a key regulator of the innate immune response and of the activation of the NLRP3-inflammasome.

The NLRC4-inflammasome is predominantly expressed in myeloid cells and is composed of an N-terminal CARD domain, which is thought to interact directly with caspase-1 [91], mediating cytokine production and the induction of cell death [92]. Activation of NLRC4 may have an important role in the defense against diverse Gram-negative bacteria, such as* Salmonella typhimurium*,* Shigella flexneri*,* Legionella pneumophila,* and* Pseudomonas aeruginosa* [93, 94], but also* Candida albicans* [95] and* Burkholderia pseudomallei*, a flagellated bacterium responsible for a tropical pneumonia [96]. In experimental and in in vitro experiments, macrophages were shown to sense the cytosolic bacterial flagellin proteins with resultant caspase-1 activation in a TLR5-independent fashion [97, 98].

The NLRP6-inflammasome has also been associated with intestinal inflammation, basically in experimental studies. For example, in NLRP6 deficient mice, the exacerbation of chemically induced colitis has been linked to the inability of repairing the injured epithelium [99]. Moreover, cohousing experiments demonstrated that the colitogenic microbiota could be transferable to wild-type mice [100]. Importantly, the NLRP6-inflammasome was also suggested to be involved in colon tumorigenesis. In this respect, NLRP6-deficient mice were shown to develop more tumors, following chemical induction with azoxymethane-dextran sodium sulfate [101].

The NLRP12 was also shown to play a role in preventing chemically induced colitis and colon tumor associated with inflammation [102], by negatively regulating of noncanonical NF-kappa B signaling [103]. However, in contrast to NLRP6, the NLRP12 effects do not appear to be associated with the regulation of the intestinal microbiota, as shown by the inability of NLRP12 deficient mice to transmit colitogenic bacteria to wild-type mice after cohousing [100].

Taken together, the results of these studies point to the relevance of the inflammasome, regarding the innate immunity and the consequent homeostatic intestinal balance. Hence, the integration of internal and external stimuli, including stressful signals and microbial components, highlights the importance of the inflammasome, which appears to constitute a mechanistic background for intestinal inflammation and the development of inflammation-associated tumorigenesis.

## 6. Conclusion

Recent investigations have provided evidence for a conceptual change in respect of the innate immune system. At first, regarded as nonspecific, the idea of innate immunity has evolved to constitute an integrative system, connecting adaptive and innate immune responses. Therefore, currently, in addition to early sensing of pathogens and delivering and immediate response, the innate immune system is implicated in the regulation and shaping of the adaptive immune response.

The hypothesis of a defective innate immunity as the primary mechanism involved with the development of IBD has been supported for more than a decade. After the first evidence indicating the genetic association of CD with NOD2 polymorphisms, a multitude of studies have been directed towards innate immunity mechanisms in IBD pathogenesis. New members of the NLRs and TLRs have been described and their functions analyzed under the light of intestinal inflammation. The pathways regulated by NLRs and TLRs were shown to be mediated by microbial elements, and they appear to be responsible for bacterial clearance and the inflammatory response, in a time-dependent fashion. In fact, most receptors of the innate immunity present ambiguous functions, according to the dynamics of the inflammatory process.

Moreover, intracellular cascades triggered by distinct receptor families may present different levels of integration and overlapping in the intestinal mucosa, in order to deal with the challenge of simultaneously responding appropriately and protecting sufficiently.

Finally, an abnormal regulation of these signaling pathways during both the early and chronic phases of intestinal inflammation may result in a persistent inflammatory process, which may underlie the pathogenesis of IBD and of the inflammation-associated colorectal cancer.
